# The genome sequence of *Inga leiocalycina* Benth.

**DOI:** 10.12688/wellcomeopenres.23131.1

**Published:** 2024-10-17

**Authors:** Rowan J. Schley, R. Toby Pennington, Alex D. Twyford, Kyle G. Dexter, Catherine Kidner, Todd P. Michael, Royal Botanic Garden Edinburgh Genome Acquisition Lab, Plant Genome Sizing collective, Wellcome Sanger Institute Tree of Life Management, Samples and Laboratory team, Wellcome Sanger Institute Scientific Operations: Sequencing Operations, Wellcome Sanger Institute Tree of Life Core Informatics team, Tree of Life Core Informatics collective

**Affiliations:** 1 University of Exeter, Exeter, England, UK; 2 Royal Botanic Garden Edinburgh, Edinburgh, Scotland, UK; 3 The University of Edinburgh, Edinburgh, Scotland, UK; 4 University of Turin, Turin, Italy; 5 Salk Institute for Biological Studies, La Jolla, California, USA; 6 University of California San Diego, San Diego, California, USA; 7 San Diego Botanical Garden, San Diego, California, USA

**Keywords:** Inga leiocalycina, genome sequence, chromosomal, Fabales

## Abstract

We present a genome assembly from an individual of *Inga leiocalycina* (Streptophyta; Magnoliopsida; Fabales; Fabaceae). The genome sequence has a total length of 948.00 megabases. Most of the assembly is scaffolded into 13 chromosomal pseudomolecules. The assembled mitochondrial genome sequences have lengths of 1,019.42 and 98.74 kilobases, and the plastid genome assembly is 175.51 kb long. Gene annotation of the nuclear genome assembly on Ensembl identified 33,457 protein-coding genes.

## Species taxonomy

Eukaryota; Viridiplantae; Streptophyta; Streptophytina; Embryophyta; Tracheophyta; Euphyllophyta; Spermatophyta; Magnoliopsida; Mesangiospermae; eudicotyledons; Gunneridae; Pentapetalae; rosids; fabids; Fabales; Fabaceae; Caesalpinioideae; mimosoid clade; Ingeae; *Inga*; *Inga leiocalycina* Benth. (NCBI:txid486065).

## Background

*Inga* Mill. (Fabaceae) is a characteristic component of the species-rich neotropical flora, and is ubiquitous in the rainforests of the tropical Americas. *Inga* typifies the rapid evolutionary radiations that generated most neotropical tree diversity, exhibiting the highest diversification rate of any tree genus in the Amazon ( Baker *et al.*, 2014; Richardson *et al.*, 2001). *Inga leiocalycina* Benth. is a tree species reaching 35m in height that is widespread in tropical American rainforests, both east and west of the Andes. This species ranges from Southern Mexico to Bolivia and Amazonian Brazil, occupying an elevational range of around 0-1000m above sea level ( Pennington, 1997). *Inga leiocalycina* tolerates a relatively wide range of rainfall conditions, ranging from the perma-wet rainforest of northwestern Colombia to the seasonally dry climate of Ecuador’s Pacific coast. *Inga leiocalycina* is also notable for having apparent edaphic ‘ecotypes’ in the Amazon, where populations on rich, bottomland soils differ morphologically from those on poorer, upland soils ( Dexter *et al.*, 2010).

Trees in tropical rainforests are subject to relentless insect herbivory, and so *Inga leiocalycina* has evolved a range of defensive strategies to deter herbivores. Specifically, *I. leiocalycina* possesses extra-floral nectaries on its leaf midribs for attracting ants that defend the plant against herbivores ( Kursar *et al.*, 2009), as well as defending itself chemically by producing a range of compounds, primarily flavan3ol_polymers, in its young leaves ( Forrister *et al.*, 2023). *Inga* species are widely used for agroforestry and ecosystem restoration due to their ability to fix nitrogen, in addition to their utility as food and forage crops ( Pennington, 1997). The fruits of *Inga leiocalycina* are edible thanks to the sweet, white sarcotesta surrounding the seeds, and are gathered from the wild as food ( López Diago & García Castro, 2021), but are not as widely used or cultivated as other *Inga* species (e.g. *Inga edulis* or *I. macrophylla*). The *Inga leiocalycina* sample sequenced here, originally collected from Manabí in western Ecuador but grown at RBGE, was a diploid (2 *n*=2 *x*=26) as is typical for the species ( Hanson, 1995).

Here we present one of three chromosomally complete, annotated genome sequences for *Inga* which are the first for the genus. We believe the *Inga leiocalycina* genome will provide an important resource for future work, given the prominent role of *Inga* in studies examining the ecology and evolution of tropical rainforest floras. Potential research themes could include assessing the role of herbivory and chemical defence in driving genomic divergence and speciation in tropical trees, building on previous work (e.g. Endara *et al.*, 2018).

## Genome sequence report

**Figure 1.**
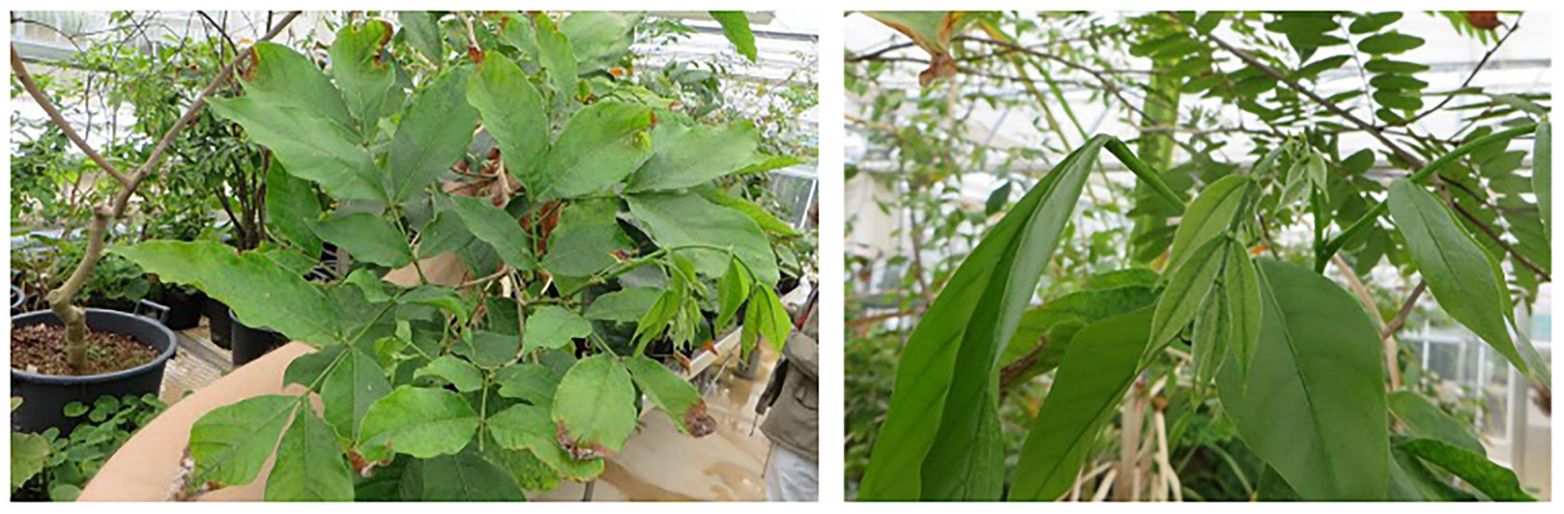
Photograph of the *Inga leiocalycina* (drIngLeio1) specimen used for genome sequencing.

The sequenced genome is of an *Inga leiocalycina* specimen (drIngLeio1, Figure 1). Using flow cytometry of leaf tissue, the genome size (1C-value) was estimated as 1.17 pg, equivalent to 1,150 Mb. The genome was sequenced using Pacific Biosciences single-molecule HiFi long reads, generating a total of 30.30 Gb (gigabases) from 2.90 million reads, providing approximately 29-fold coverage. Primary assembly contigs were scaffolded with chromosome conformation Hi-C data, which produced 117.73 Gb from 779.69 million reads, yielding an approximate coverage of 124-fold. Specimen and sequencing information is summarised in Table 1.

**Table 1.** Specimen and sequencing data for *Inga leiocalycina*.

Project information			
**Study title**	Inga leiocalycina		
**Umbrella BioProject**	PRJEB64758		
**Species**	*Inga leiocalycina*		
**BioSample**	SAMEA111531407		
**NCBI taxonomy ID**	486065		
Specimen information			
**Technology**	**ToLID**	**BioSample accession**	**Organism part**
**PacBio long read sequencing**	drIngLeio1	SAMEA111531416	Leaf
**Hi-C sequencing**	drIngLeio1	SAMEA111531409	Leaf
**RNA sequencing**	drIngLeio2	SAMEA113598545	leaf
Sequencing information			
**Platform**	**Run accession**	**Read count**	**Base count (Gb)**
**Hi-C Illumina NovaSeq 6000**	ERR11814135	7.80e+08	117.73
**PacBio Sequel IIe**	ERR11809161	2.90e+06	30.3
**RNA Illumina NovaSeq 6000**	ERR12321232	7.00e+07	10.57

**Figure 2.**
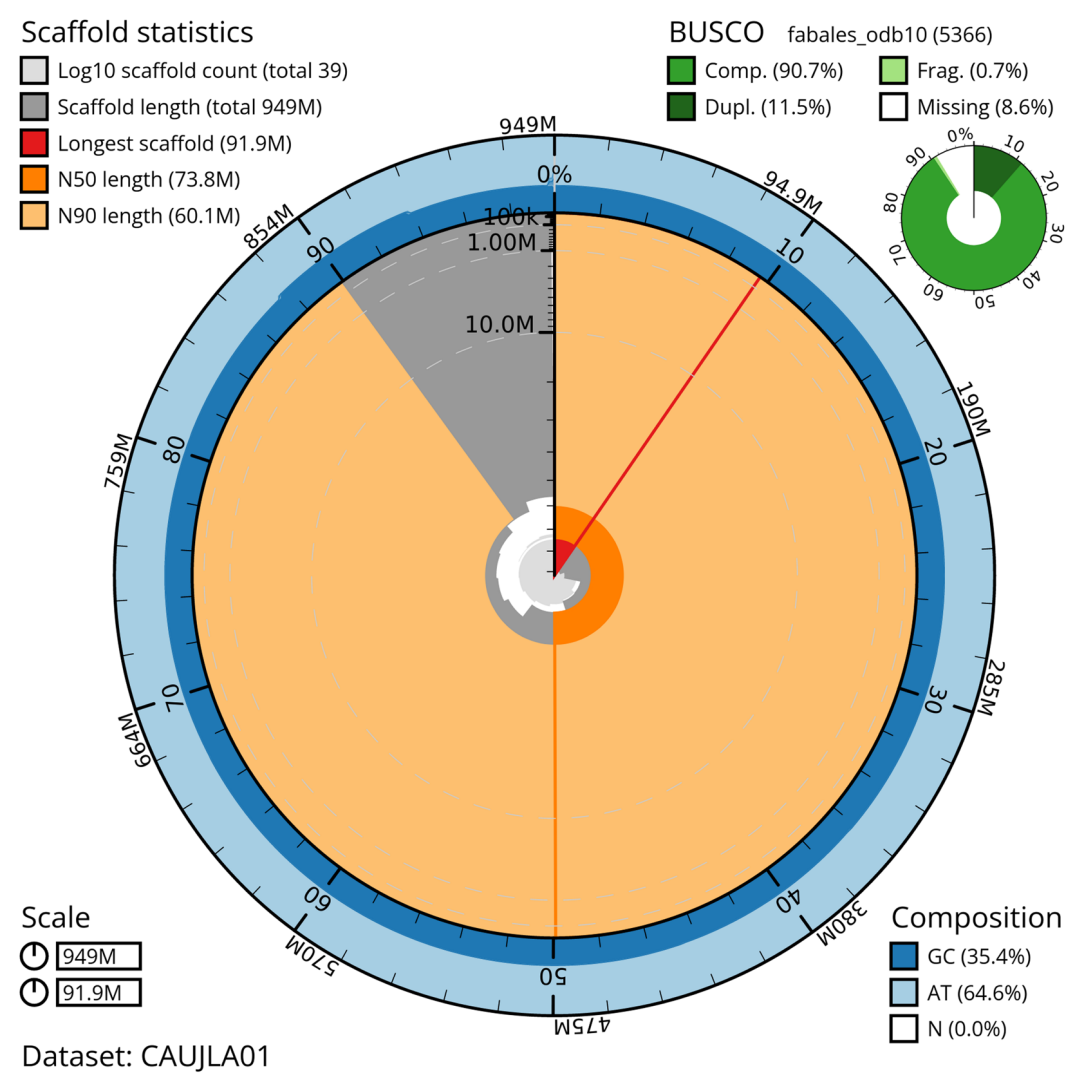
Genome assembly of *Inga leiocalycina*, drIngLeio1.1: metrics. The BlobToolKit snail plot shows N50 metrics and BUSCO gene completeness. The main plot is divided into 1,000 size-ordered bins around the circumference with each bin representing 0.1% of the 949,261,181 bp assembly. The distribution of scaffold lengths is shown in dark grey with the plot radius scaled to the longest scaffold present in the assembly (91,898,272 bp, shown in red). Orange and pale-orange arcs show the N50 and N90 scaffold lengths (73,756,888 and 60,100,043 bp), respectively. The pale grey spiral shows the cumulative scaffold count on a log scale with white scale lines showing successive orders of magnitude. The blue and pale-blue area around the outside of the plot shows the distribution of GC, AT and N percentages in the same bins as the inner plot. A summary of complete, fragmented, duplicated and missing BUSCO genes in the fabales_odb10 set is shown in the top right. An interactive version of this figure is available at https://blobtoolkit.genomehubs.org/view/CAUJLA01/dataset/CAUJLA01/snail.

**Figure 3.**
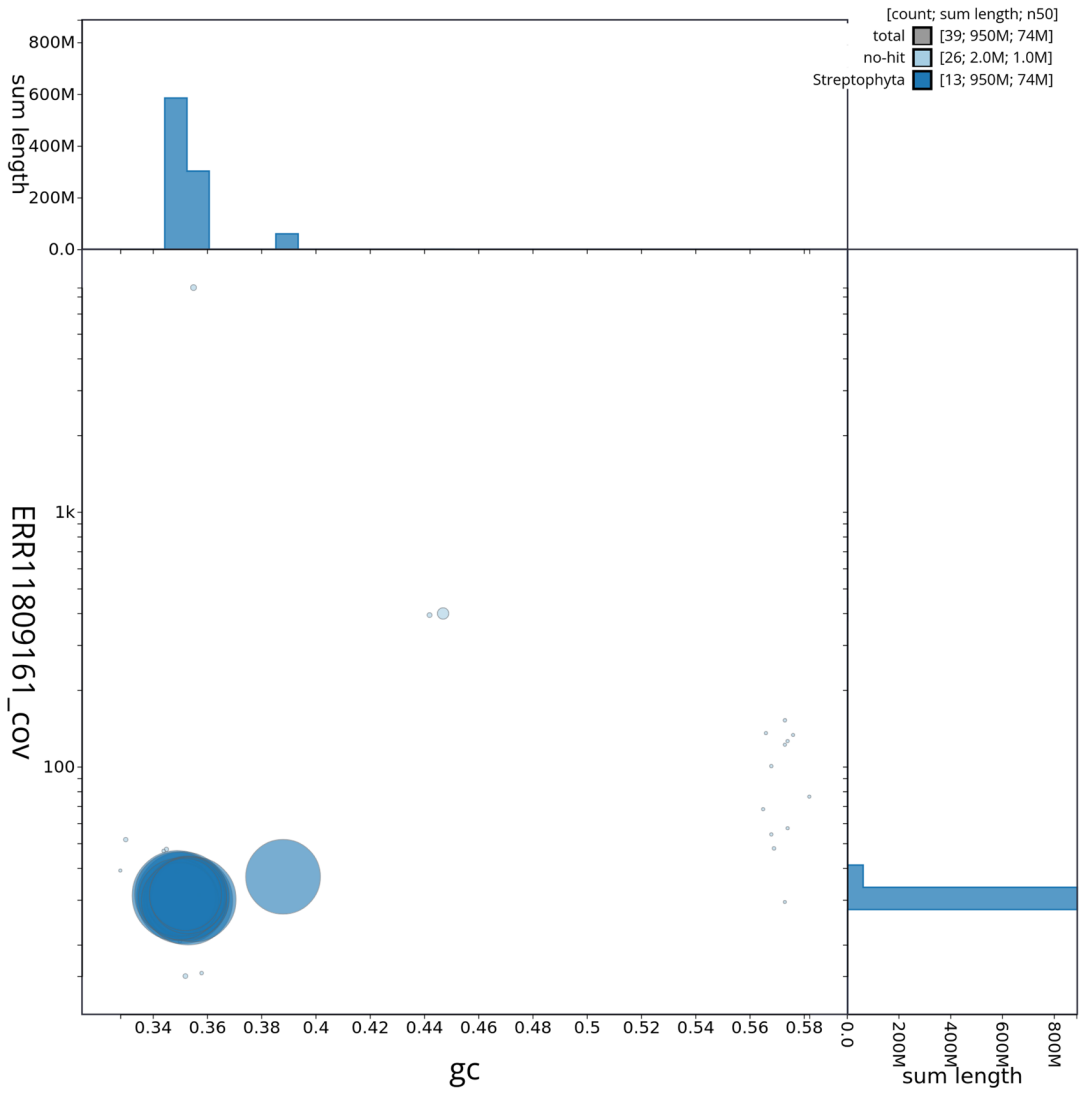
Genome assembly of *Inga leiocalycina*, drIngLeio1.1: BlobToolKit GC-coverage plot. Sequences are coloured by phylum. Circles are sized in proportion to sequence length. Histograms show the distribution of sequence length sum along each axis. An interactive version of this figure is available at https://blobtoolkit.genomehubs.org/view/CAUJLA01/dataset/CAUJLA01/blob.

**Figure 4.**
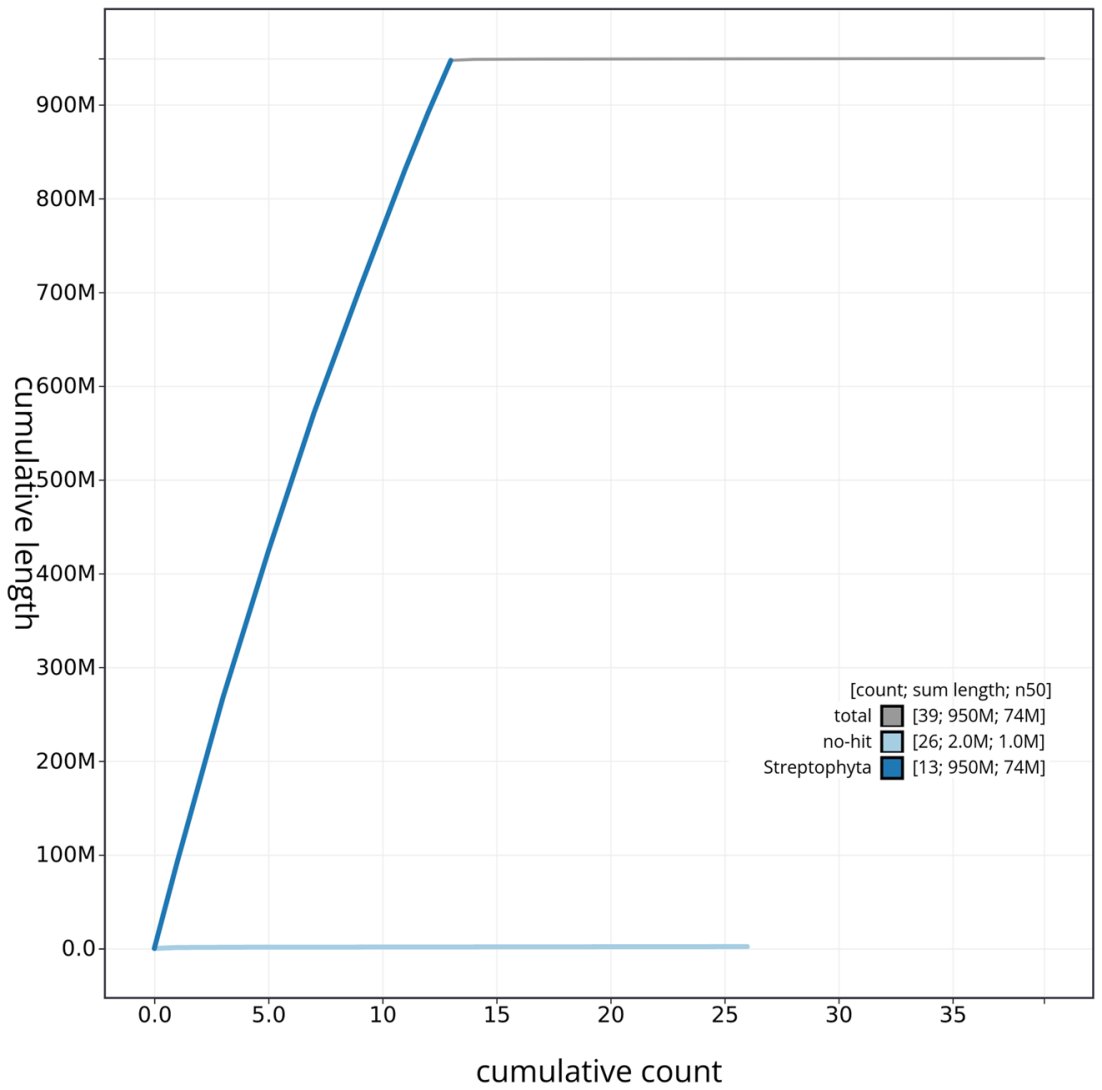
Genome assembly of *Inga leiocalycina* drIngLeio1.1: BlobToolKit cumulative sequence plot. The grey line shows cumulative length for all sequences. Coloured lines show cumulative lengths of sequences assigned to each phylum using the buscogenes taxrule. An interactive version of this figure is available at https://blobtoolkit.genomehubs.org/view/CAUJLA01/dataset/CAUJLA01/cumulative.

**Figure 5.**
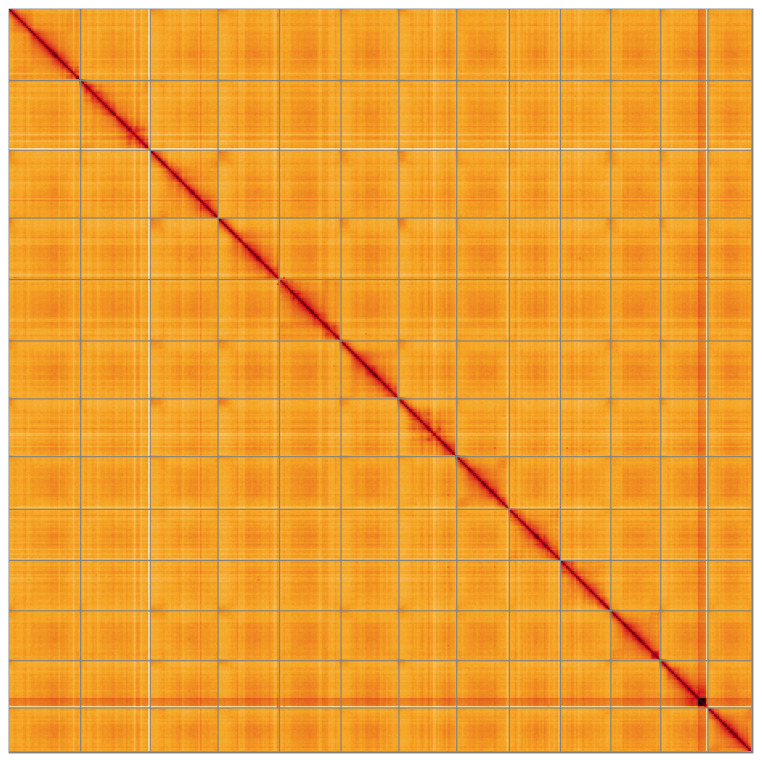
Genome assembly of *Inga leiocalycina*, drIngLeio1.1: Hi-C contact map of the drIngLeio1.1 assembly, visualised using HiGlass. Chromosomes are shown in order of size from left to right and top to bottom. An interactive version of this figure may be viewed at https://genome-note-higlass.tol.sanger.ac.uk/l/?d=abMPGhQDQSOODVsPWKcBtw.

Manual assembly curation corrected 100 missing joins or mis-joins and 25 haplotypic duplications, reducing the assembly length by 2.52%, and decreasing the scaffold N50 by 18.54%. The final assembly has a total length of 948.00 Mb in 36 sequence scaffolds with a scaffold N50 of 73.8 Mb ( Table 2) with 362 gaps. The snail plot in Figure 2 provides a summary of the assembly statistics, while the distribution of assembly scaffolds on GC proportion and coverage is shown in Figure 3. The cumulative assembly plot in Figure 4 shows curves for subsets of scaffolds assigned to different phyla. Most (99.78%) of the assembly sequence was assigned to 13 chromosomal-level scaffolds. Chromosome-scale scaffolds confirmed by the Hi-C data are named in order of size ( Figure 5; Table 3). The order and orientation of contigs along Chromosome 12 between 49.5 Mb and 58.3 Mb is uncertain. While not fully phased, the assembly deposited is of one haplotype. Contigs corresponding to the second haplotype have also been deposited. The mitochondrial and plastid genomes were also assembled and can be found as contigs within the multifasta file of the genome submission.

**Table 2.** Genome assembly data for *Inga leiocalycina*, drIngLeio1.1.

Genome assembly		
Assembly name	drIngLeio1.1	
Assembly accession	GCA_963242795.1	
*Accession of alternate haplotype*	*GCA_963242585.1*	
Span (Mb)	948.00	
Number of contigs	401	
Contig N50 length (Mb)	5.6	
Number of scaffolds	36	
Scaffold N50 length (Mb)	73.8	
Longest scaffold (Mb)	91.9	
Assembly metrics *		*Benchmark*
Consensus quality (QV)	64.6	*≥ 50*
*k*-mer completeness	100.0%	*≥ 95%*
BUSCO **	C:90.7%[S:79.2%,D:11.5%], F:0.7%,M:8.6%,n:5,366	*C ≥ 95%*
Percentage of assembly mapped to chromosomes	99.78%	*≥ 95%*
Organelles	Mitochondrial genome: 1,019.42 kb and 98.74 kb; plastid genome: 175.51 kb	*complete single alleles*
Genome annotation at Ensembl		
Number of protein-coding genes	33,457	
Number of non-coding genes	14,611	
Number of gene transcripts	69,098	

* Assembly metric benchmarks are adapted from column VGP-2020 of “Table 1: Proposed standards and metrics for defining genome assembly quality” from Rhie *et al.* (2021). ** BUSCO scores based on the fabales_odb10 BUSCO set using version 5.4.3. C = complete [S = single copy, D = duplicated], F = fragmented, M = missing, n = number of orthologues in comparison. A full set of BUSCO scores is available at https://blobtoolkit.genomehubs.org/view/CAUJLA01/dataset/CAUJLA01/busco.

**Table 3.** Chromosomal pseudomolecules in the genome assembly of *Inga leiocalycina*, drIngLeio1.

INSDC accession	Name	Length (Mb)	GC%
OY725320.1	1	91.9	35.0
OY725321.1	2	88.61	35.0
OY725322.1	3	86.41	35.5
OY725323.1	4	78.49	35.5
OY725324.1	5	78.27	35.0
OY725325.1	6	73.76	35.5
OY725326.1	7	73.71	35.0
OY725327.1	8	67.02	35.0
OY725328.1	9	65.06	35.0
OY725329.1	10	64.34	35.0
OY725330.1	11	63.58	35.5
OY725331.1	12	60.1	39.0
OY725332.1	13	56.0	35.0
OY725335.1	Pltd	0.18	35.5
OY725333.1	MT1	1.02	44.5
OY725334.1	MT2	0.1	44.0

The estimated Quality Value (QV) of the final assembly is 64.6 with *k*-mer completeness of 100.0%, and the assembly has a BUSCO v5.4.3 completeness of 90.7% (single = 79.2%, duplicated = 11.5%), using the fabales_odb10 reference set ( *n* = 5,366).

Metadata for specimens, BOLD barcode results, spectra estimates, sequencing runs, contaminants and pre-curation assembly statistics are given at https://links.tol.sanger.ac.uk/species/486065.

## Genome annotation report

The *Inga leiocalycina* genome assembly (GCA_963242795.1) was annotated at the European Bioinformatics Institute (EBI) on Ensembl Rapid Release. The resulting annotation includes 69,098 transcribed mRNAs from 33,457 protein-coding and 14,611 non-coding genes ( Table 2; https://rapid.ensembl.org/Inga_leiocalycina_GCA_963242795.1/Info/Index). The average transcript length is 3,476.11. There are 1.44 coding transcripts per gene and 4.78 exons per transcript.

## Methods

### Sample acquisition and nucleic acid extraction

A specimen of *Inga leiocalycina* (specimen ID SAN2000548, ToLID drIngLeio1) was collected on 2021-09-09 from the wet tropics glasshouse at the Royal Botanic Garden Edinburgh, Scotland, UK. The specimen used for RNA sequencing (specimen ID SAN20001664, ToLID drIngLeio2) was collected from the same individual on 2023-05-31. The specimens were collected by Rowan Schley (University of Exeter). The original individual was collected in Manabí, Ecuador in 1993 under the collector number ‘T.D. Pennington 13822’ and identified by Terence D. Pennington (Royal Botanic Gardens Kew). The herbarium voucher associated with the sequenced plant is RBGE:BROWP2035 and is deposited in the herbarium of the Royal Botanic Garden Edinburgh (Herbarium code: E).

The workflow for high molecular weight (HMW) DNA extraction at the Wellcome Sanger Institute (WSI) Tree of Life Core Laboratory includes a sequence of core procedures: sample preparation; sample homogenisation, DNA extraction, fragmentation, and clean-up. Leaf tissue of the drIngLeio1 sample was weighed and dissected on dry ice ( Jay *et al.*, 2023), and cryogenically disrupted using the Covaris cryoPREP ^®^ Automated Dry Pulverizer ( Narváez-Gómez *et al.*, 2023). HMW DNA was extracted using the Manual Plant MagAttract v4 protocol ( Jackson & Howard, 2023). HMW DNA was sheared into an average fragment size of 12–20 kb in a Megaruptor 3 system ( Bates *et al.*, 2023). Sheared DNA was purified by solid-phase reversible immobilisation ( Oatley *et al.*, 2023): in brief, the method employs AMPure PB beads to eliminate shorter fragments and concentrate the DNA. The concentration of the sheared and purified DNA was assessed using a Nanodrop spectrophotometer and Qubit Fluorometer and Qubit dsDNA High Sensitivity Assay kit. Fragment size distribution was evaluated by running the sample on the FemtoPulse system.

RNA was extracted from leaf tissue of drIngLeio2 in the Tree of Life Laboratory at the WSI using the RNA Extraction: Automated MagMax™ *mir*Vana protocol ( do Amaral *et al.*, 2023). The RNA concentration was assessed using a Nanodrop spectrophotometer and a Qubit Fluorometer using the Qubit RNA Broad-Range Assay kit. Analysis of the integrity of the RNA was done using the Agilent RNA 6000 Pico Kit and Eukaryotic Total RNA assay.

Protocols developed by the WSI Tree of Life core laboratory are publicly available on protocols.io ( Denton *et al.*, 2023).

### Sequencing

Pacific Biosciences HiFi circular consensus DNA sequencing libraries were constructed according to the manufacturers’ instructions. Poly(A) RNA-Seq libraries were constructed using the NEB Ultra II RNA Library Prep kit. DNA and RNA sequencing was performed by the Scientific Operations core at the WSI on Pacific Biosciences Sequel IIe (HiFi) and Illumina NovaSeq 6000 (RNA-Seq) instruments. Hi-C data were also generated from leaf tissue of drIngLeio1 using the Arima-HiC v2 kit. The Hi-C sequencing was performed using paired-end sequencing with a read length of 150 bp on the Illumina NovaSeq 6000 instrument.

### Genome assembly, curation and evaluation

***Assembly***

The original assembly of HiFi reads was performed using Hifiasm ( Cheng *et al.*, 2021) with the --primary option. Haplotypic duplications were identified and removed with purge_dups ( Guan *et al.*, 2020). Hi-C reads were further mapped with bwa-mem2 ( Vasimuddin *et al.*, 2019) to the primary contigs, which were further scaffolded using the provided Hi-C data ( Rao *et al.*, 2014) in YaHS ( Zhou *et al.*, 2023) using the --break option. Scaffolded assemblies were evaluated using Gfastats ( Formenti *et al.*, 2022), BUSCO ( Manni *et al.*, 2021) and MERQURY.FK ( Rhie *et al.*, 2020). The organelle genomes were assembled using OATK ( Zhou, 2023).

***Curation***

The assembly was decontaminated using the Assembly Screen for Cobionts and Contaminants (ASCC) pipeline (article in preparation). Manual curation was primarily conducted using PretextView ( Harry, 2022), with additional insights provided by JBrowse2 ( Diesh *et al.*, 2023) and HiGlass ( Kerpedjiev *et al.*, 2018). Scaffolds were visually inspected and corrected as described by Howe *et al*. (2021). Any identified contamination, missed joins, and mis-joins were corrected, and duplicate sequences were tagged and removed. The process is documented at https://gitlab.com/wtsi-grit/rapid-curation (article in preparation).

***Evaluation of final assembly***

A Hi-C map for the final assembly was produced using bwa-mem2 ( Vasimuddin *et al.*, 2019) in the Cooler file format ( Abdennur & Mirny, 2020). To assess the assembly metrics, the *k*-mer completeness and QV consensus quality values were calculated in Merqury ( Rhie *et al.*, 2020). This work was done using the “sanger-tol/readmapping” ( Surana *et al.*, 2023a) and “sanger-tol/genomenote” ( Surana *et al.*, 2023b) pipelines. The genome readmapping pipelines were developed using the nf-core tooling ( Ewels *et al.*, 2020), use MultiQC ( Ewels *et al.*, 2016), and make extensive use of the Conda package manager, the Bioconda initiative ( Grüning *et al.*, 2018), the Biocontainers infrastructure ( da Veiga Leprevost *et al.*, 2017), and the Docker ( Merkel, 2014) and Singularity ( Kurtzer *et al.*, 2017) containerisation solutions. The genome was also analysed within the BlobToolKit environment ( Challis *et al.*, 2020) and BUSCO scores ( Manni *et al.*, 2021) were calculated.

Table 4 contains a list of relevant software tool versions and sources.

**Table 4.** Software tools: versions and sources.

Software tool	Version	Source
BlobToolKit	4.2.1	https://github.com/blobtoolkit/blobtoolkit
BUSCO	5.3.2	https://gitlab.com/ezlab/busco
bwa-mem2	2.2.1	https://github.com/bwa-mem2/bwa-mem2
Cooler	0.8.11	https://github.com/open2c/cooler
Gfastats	1.3.6	https://github.com/vgl-hub/gfastats
Hifiasm	0.19.5-r587	https://github.com/chhylp123/hifiasm
HiGlass	1.11.6	https://github.com/higlass/higlass
Merqury	MerquryFK	https://github.com/thegenemyers/MERQURY.FK
OATK	0.9	https://github.com/c-zhou/oatk
PretextView	0.2	https://github.com/wtsi-hpag/PretextView
purge_dups	1.2.3	https://github.com/dfguan/purge_dups
sanger-tol/genomenote	v1.0	https://github.com/sanger-tol/genomenote
sanger-tol/readmapping	1.1.0	https://github.com/sanger-tol/readmapping/tree/1.1.0
YaHS	1.1a.2	https://github.com/c-zhou/yahs

### Wellcome Sanger Institute – Legal and Governance

The materials that have contributed to this genome note have been supplied by a Darwin Tree of Life Partner. The submission of materials by a Darwin Tree of Life Partner is subject to the **‘Darwin Tree of Life Project Sampling Code of Practice’**, which can be found in full on the Darwin Tree of Life website here. By agreeing with and signing up to the Sampling Code of Practice, the Darwin Tree of Life Partner agrees they will meet the legal and ethical requirements and standards set out within this document in respect of all samples acquired for, and supplied to, the Darwin Tree of Life Project.

Further, the Wellcome Sanger Institute employs a process whereby due diligence is carried out proportionate to the nature of the materials themselves, and the circumstances under which they have been/are to be collected and provided for use. The purpose of this is to address and mitigate any potential legal and/or ethical implications of receipt and use of the materials as part of the research project, and to ensure that in doing so we align with best practice wherever possible. The overarching areas of consideration are:

•   Ethical review of provenance and sourcing of the material

•   Legality of collection, transfer and use (national and international)

Each transfer of samples is further undertaken according to a Research Collaboration Agreement or Material Transfer Agreement entered into by the Darwin Tree of Life Partner, Genome Research Limited (operating as the Wellcome Sanger Institute), and in some circumstances other Darwin Tree of Life collaborators.

## Data Availability

European Nucleotide Archive: *Inga leiocalycina*. Accession number PRJEB64758; https://identifiers.org/ena.embl/PRJEB64758 ( Wellcome Sanger Institute, 2023). The genome sequence is released openly for reuse. The *Inga leiocalycina* genome sequencing initiative is part of the Darwin Tree of Life (DToL) project. All raw sequence data and the assembly have been deposited in INSDC databases. Raw data and assembly accession identifiers are reported in Table 1.

## Author information

Members of the Royal Botanic Garden Edinburgh Genome Acquisition Lab are listed here: https://doi.org/10.5281/zenodo.4786682.

Members of the Plant Genome Sizing collective are listed here: https://doi.org/10.5281/zenodo.7994306.

Members of the Wellcome Sanger Institute Tree of Life Management, Samples and Laboratory team are listed here: https://doi.org/10.5281/zenodo.12162482.

Members of Wellcome Sanger Institute Scientific Operations: Sequencing Operations are listed here: https://doi.org/10.5281/zenodo.12165051.

Members of the Wellcome Sanger Institute Tree of Life Core Informatics team are listed here: https://doi.org/10.5281/zenodo.12160324.

Members of the Tree of Life Core Informatics collective are listed here: https://doi.org/10.5281/zenodo.12205391.

## References

[ref-1] Abdennur N Mirny LA : Cooler: scalable storage for Hi-C data and other genomically labeled arrays. *Bioinformatics.* 2020;36(1):311–316. 10.1093/bioinformatics/btz540 31290943 PMC8205516

[ref-2] Baker TR Pennington RT Magallon S : Fast demographic traits promote high diversification rates of Amazonian trees. *Ecol Lett.* 2014;17(5):527–536. 10.1111/ele.12252 24589190 PMC4285998

[ref-3] Bates A Clayton-Lucey I Howard C : Sanger Tree of Life HMW DNA fragmentation: diagenode Megaruptor ^®^3 for LI PacBio. *protocols.io.* 2023. 10.17504/protocols.io.81wgbxzq3lpk/v1

[ref-4] Challis R Richards E Rajan J : BlobToolKit – interactive quality assessment of genome assemblies. *G3 (Bethesda).* 2020;10(4):1361–1374. 10.1534/g3.119.400908 32071071 PMC7144090

[ref-5] Cheng H Concepcion GT Feng X : Haplotype-resolved *de novo* assembly using phased assembly graphs with hifiasm. *Nat Methods.* 2021;18(2):170–175. 10.1038/s41592-020-01056-5 33526886 PMC7961889

[ref-6] da Veiga Leprevost F Grüning BA Alves Aflitos S : BioContainers: an open-source and community-driven framework for software standardization. *Bioinformatics.* 2017;33(16):2580–2582. 10.1093/bioinformatics/btx192 28379341 PMC5870671

[ref-7] Denton A Yatsenko H Jay J : Sanger Tree of Life wet laboratory protocol collection. *protocols.io.* 2023. 10.17504/protocols.io.8epv5xxy6g1b/v1

[ref-8] Dexter KG Pennington TD Cunningham CW : Using DNA to assess errors in tropical tree identifications: how often are ecologists wrong and when does it matter? *Ecol Monogr.* 2010;80(2):267–286. 10.1890/09-0267.1

[ref-9] Diesh C Stevens GJ Xie P : JBrowse 2: a modular genome browser with views of synteny and structural variation. *Genome Biol.* 2023;24(1): 74. 10.1186/s13059-023-02914-z 37069644 PMC10108523

[ref-10] do Amaral RJV Bates A Denton A : Sanger Tree of Life RNA extraction: automated MagMax ^TM^ mirVana. *protocols.io.* 2023. 10.17504/protocols.io.6qpvr36n3vmk/v1

[ref-11] Endara MJ Coley PD Wiggins NL : Chemocoding as an identification tool where morphological- and DNA-based methods fall short: *Inga* as a case study. *New Phytol.* 2018;218(2):847–858. 10.1111/nph.15020 29436716

[ref-13] Ewels P Magnusson M Lundin S : MultiQC: summarize analysis results for multiple tools and samples in a single report. *Bioinformatics.* 2016;32(19):3047–3048. 10.1093/bioinformatics/btw354 27312411 PMC5039924

[ref-12] Ewels PA Peltzer A Fillinger S : The nf-core framework for community-curated bioinformatics pipelines. *Nat Biotechnol.* 2020;38(3):276–278. 10.1038/s41587-020-0439-x 32055031

[ref-14] Formenti G Abueg L Brajuka A : Gfastats: conversion, evaluation and manipulation of genome sequences using assembly graphs. *Bioinformatics.* 2022;38(17):4214–4216. 10.1093/bioinformatics/btac460 35799367 PMC9438950

[ref-15] Forrister DL Endara MJ Soule AJ : Diversity and divergence: evolution of secondary metabolism in the tropical tree genus *Inga*. *New Phytol.* 2023;237(2):631–642. 10.1111/nph.18554 36263711

[ref-16] Grüning B Dale R Sjödin A : Bioconda: sustainable and comprehensive software distribution for the life sciences. *Nat Methods.* 2018;15(7):475–476. 10.1038/s41592-018-0046-7 29967506 PMC11070151

[ref-17] Guan D McCarthy SA Wood J : Identifying and removing haplotypic duplication in primary genome assemblies. *Bioinformatics.* 2020;36(9):2896–2898. 10.1093/bioinformatics/btaa025 31971576 PMC7203741

[ref-18] Hanson L : Some new chromosome counts in the genus *Inga* (Leguminosae: Mimosoideae). *Kew Bull.* 1995;50(4):801–804. 10.2307/4110243

[ref-19] Harry E : PretextView (Paired REad TEXTure Viewer): a desktop application for viewing pretext contact maps.2022. Reference Source

[ref-20] Howe K Chow W Collins J : Significantly improving the quality of genome assemblies through curation. *GigaScience.* 2021;10(1): giaa153. 10.1093/gigascience/giaa153 33420778 PMC7794651

[ref-21] Jackson B Howard C : Sanger Tree of Life HMW DNA extraction: manual plant MagAttract v.4. *protocols.io.* 2023; [Accessed 8 July 2024]. 10.17504/protocols.io.261ged5k7v47/v1

[ref-40] Jay J Yatsenko H Narváez-Gómez JP : Sanger Tree of Life sample preparation: triage and dissection. *protocols.io.* 2023. 10.17504/protocols.io.x54v9prmqg3e/v1

[ref-22] Kerpedjiev P Abdennur N Lekschas F : HiGlass: web-based visual exploration and analysis of genome interaction maps. *Genome Biol.* 2018;19(1): 125. 10.1186/s13059-018-1486-1 30143029 PMC6109259

[ref-23] Kursar TA Dexter KG Lokvam J : The evolution of antiherbivore defenses and their contribution to species coexistence in the tropical tree genus Inga. *Proc Natl Acad Sci U S A.* 2009;106(43):18073–8. 10.1073/pnas.0904786106 19805183 PMC2775284

[ref-24] Kurtzer GM Sochat V Bauer MW : Singularity: scientific containers for mobility of compute. *PLoS One.* 2017;12(5): e0177459. 10.1371/journal.pone.0177459 28494014 PMC5426675

[ref-25] López Diago D García Castro NJ : Wild edible fruits of Colombia: diversity and use prospects. [Frutos silvestres comestibles de Colombia: diversidad y perspectivas de uso. *Biota Colombiana.* 2021;22(2):16–55. 10.21068/c2021.v22n02a02

[ref-26] Manni M Berkeley MR Seppey M : BUSCO update: novel and streamlined workflows along with broader and deeper phylogenetic coverage for scoring of eukaryotic, prokaryotic, and viral genomes. *Mol Biol Evol.* 2021;38(10):4647–4654. 10.1093/molbev/msab199 34320186 PMC8476166

[ref-27] Merkel D : Docker: lightweight Linux containers for consistent development and deployment. *Linux J.* 2014;2014(239):2. [Accessed 2 April 2024]. Reference Source

[ref-41] Narváez-Gómez JP Mbye H Oatley G : Sanger Tree of Life sample homogenisation: Covaris cryoPREP® automated dry pulverizer. *protocols.io.* 2023. 10.17504/protocols.io.eq2lyjp5qlx9/v1

[ref-28] Oatley G Sampaio F Howard C : Sanger Tree of Life fragmented DNA clean up: automated SPRI. *protocols.io.* 2023. 10.17504/protocols.io.q26g7p1wkgwz/v1

[ref-29] Pennington TD : The genus inga: botany. London: Royal Botanic Gardens, Kew,1997. Reference Source

[ref-30] Rao SSP Huntley MH Durand NC : A 3D map of the human genome at kilobase resolution reveals principles of chromatin looping. *Cell.* 2014;159(7):1665–1680. 10.1016/j.cell.2014.11.021 25497547 PMC5635824

[ref-31] Rhie A McCarthy SA Fedrigo O : Towards complete and error-free genome assemblies of all vertebrate species. *Nature.* 2021;592(7856):737–746. 10.1038/s41586-021-03451-0 33911273 PMC8081667

[ref-32] Rhie A Walenz BP Koren S : Merqury: reference-free quality, completeness, and phasing assessment for genome assemblies. *Genome Biol.* 2020;21(1): 245. 10.1186/s13059-020-02134-9 32928274 PMC7488777

[ref-33] Richardson JE Pennington RT Pennington TD : Rapid diversification of a species-rich genus of neotropical rain forest trees. *Science.* 2001;293(5538):2242–2245. 10.1126/science.1061421 11567135

[ref-34] Surana P Muffato M Qi G : sanger-tol/readmapping: sanger-tol/readmapping v1.1.0 - Hebridean Black (1.1.0). *Zenodo.* 2023a. 10.5281/zenodo.7755669

[ref-35] Surana P Muffato M Sadasivan Baby C : sanger-tol/genomenote (v1.0.dev). *Zenodo.* 2023b. 10.5281/zenodo.6785935

[ref-36] Vasimuddin Md Misra S Li H : Efficient architecture-aware acceleration of BWA-MEM for multicore systems.In: *2019 IEEE International Parallel and Distributed Processing Symposium (IPDPS).*IEEE,2019;314–324. 10.1109/IPDPS.2019.00041

[ref-39] Wellcome Sanger Institute: The genome sequence of *Inga leiocalycina* Benth. European Nucleotide Archive.[dataset], accession number PRJEB64758,2023.

[ref-37] Zhou C : c-zhou/oatk: Oatk-0.1.2023. 10.5281/zenodo.7631375

[ref-38] Zhou C McCarthy SA Durbin R : YaHS: yet another Hi-C scaffolding tool. *Bioinformatics.* 2023;39(1): btac808. 10.1093/bioinformatics/btac808 36525368 PMC9848053

