# Supplementary material for: The genome sequence of Inga leiocalycina Benth
Source: Wellcome Open Res. 2024 Oct 17;9:606. [Version 1] doi: 10.12688/wellcomeopenres.23131.1 (PMC11531642; doi:10.12688/wellcomeopenres.23131.1)
Supplement: Peer review history

# Reviewer report: Reviewer response for version 1

**DOI:** 10.21956/wellcomeopenres.25471.r106021

**Published:** 2024-11-07

**Authors:** Mark Chapman

**Affiliations:** 1 University of Southampton, Southampton, UK

The article presents a genome sequence for an interesting tree species (genus Inga) from South America. The justification for choice is sound and I would imagine understanding more about the wide adaptation of this species is a future goal. I have just minor points:

 ‘Most’ in the abstract is a bit vague, maybe present the actual percentage, especially as it’s a very high percentage (this info is only in the main body of the ms).

 Abstract - consistently use ‘kilobases’ or ‘kb’ (or say ‘kilobases (kb)’ the first time and kb after)

 Should ‘Inga leiocalycina’ in the keywords be italicised?

 Although most readers will understand this, “There are 1.44 coding transcripts per gene and 4.78 exons per transcript.” suggests this is a fixed number and not the average, therefore please edit to state this is the average.

Are sufficient details of methods and materials provided to allow replication by others?

Yes

Is the rationale for creating the dataset(s) clearly described?

Yes

Are the datasets clearly presented in a useable and accessible format?

Yes

Are the protocols appropriate and is the work technically sound?

Yes

Reviewer Expertise:

Plant genomics, population genetics

I confirm that I have read this submission and believe that I have an appropriate level of expertise to confirm that it is of an acceptable scientific standard.

# Reviewer report: Reviewer response for version 1

**DOI:** 10.21956/wellcomeopenres.25471.r106013

**Published:** 2024-11-02

**Authors:** Zhang Rong

**Affiliations:** 1 Chinese Academy of Sciences, Kunming Institute of Botany, Kunming, Yunnan, China

This manuscript presents a genome assembly of *Inga leiocalycina* (Fabaceae), with a total length of 948.00 Mb across 36 sequence scaffolds and a scaffold N50 of 73.8 Mb. Nearly all (99.78%) of the sequence was organized into 13 chromosomal-level scaffolds. Ensembl gene annotation identified 33,457 protein-coding genes in the nuclear genome. This assembly provides valuable insights for studies on the genomics and phylogenomics of the species-rich Fabaceae family. The sequencing rationale and supporting information are clearly outlined, and the methods and materials contain sufficient detail for replication.

Are sufficient details of methods and materials provided to allow replication by others?

Yes

Is the rationale for creating the dataset(s) clearly described?

Yes

Are the datasets clearly presented in a useable and accessible format?

Yes

Are the protocols appropriate and is the work technically sound?

Yes

Reviewer Expertise:

My expertise is phylogenomics of Fabaceae.

I confirm that I have read this submission and believe that I have an appropriate level of expertise to confirm that it is of an acceptable scientific standard.

# Reviewer report: Reviewer response for version 1

**DOI:** 10.21956/wellcomeopenres.25471.r106003

**Published:** 2024-11-01

**Authors:** Deniz Aygören Uluer

**Affiliations:** 1 Ahi Evran University, Kirşehir, Turkey

This is a well written study about the genome sequencing of *Inga leiocalycina*. I only have a few suggestions:

 -Once writing the full name of the species, the name can be written as "I. leiocalycina"

 -The species is also notable for having apparent edaphic ‘ecotypes’ in the Amazon.... (repeating the species name over and over does not sound well)

Are sufficient details of methods and materials provided to allow replication by others?

Yes

Is the rationale for creating the dataset(s) clearly described?

Yes

Are the datasets clearly presented in a useable and accessible format?

Yes

Are the protocols appropriate and is the work technically sound?

Yes

Reviewer Expertise:

Plant systematics

I confirm that I have read this submission and believe that I have an appropriate level of expertise to confirm that it is of an acceptable scientific standard.
